# RACIAL DIFFERENCES IN CAREGIVER BURDEN AMONG BLACK/AFRICAN AMERICAN AND WHITE ALL-CAUSE CAREGIVERS

**DOI:** 10.1093/geroni/igad104.2224

**Published:** 2023-12-21

**Authors:** Maizonne Fields, Olivio Clay

**Affiliations:** The University of Alabama at Birmingham, Birmingham, Alabama, United States; University of Alabama at Birmingham, Birmingham, Alabama, United States

## Abstract

**Introduction:**

As life expectancy and the numbers of older adults in our society continue to rise, serving as a caregiver is more common. Caregivers assist with a variety of daily tasks as changes in functional status occur. The Stress Process Model informs that there are potential buffers to negative caregiver outcomes including religious commitment or involvement and cultural justifications for caregiving. Previously, research has shown that caregivers may turn to religion and cultural practices to cope with or explain their roles.

**Methods:**

This investigation examines racial differences in caregiver burden and the potential roles of caregiver religiosity and cultural justifications for caregiving as moderators of this effect. Individuals providing care for an adult age 45 and older for at least five hours per week were recruited.

**Results:**

The sample size for this study was 80 and included 49 Black/African American caregivers (61.25%) and 31 White caregivers (38.75%) between the ages of 23 and 78. Multiple regression models with age, gender, income difficulty, and race as predictors revealed that Black/African American caregivers reported lower levels of caregiver burden compared to White caregivers, p < .05. Additional interaction terms added to the model revealed that religious commitment or involvement and cultural justifications for caregiving did not moderate the effect of race on caregiver burden.

**Conclusion:**

The initial findings of this investigation may be the result of the study’s small sample size and insufficient power. Recruitment is ongoing to help identify factors to potentially explain why Black/African American caregivers report less caregiver burden.

